# High resolution iridocorneal angle imaging system by axicon lens assisted gonioscopy

**DOI:** 10.1038/srep30844

**Published:** 2016-07-29

**Authors:** Sandeep Menon Perinchery, Anant Shinde, Chan Yiu Fu, Xun Jie Jeesmond Hong, Mani Baskaran, Tin Aung, Vadakke Matham Murukeshan

**Affiliations:** 1 School of Mechanical and Aerospace Engineering, Nanyang Technological University, Singapore 639798; 2 Centre for Optical and Laser Engineering, 50 Nanyang Avenue, Singapore 639798; 3 Singapore Eye Research Institute (SERI) and Singapore National Eye Centre (SNEC), 11 Third Hospital Avenue, Singapore 168751; 4 Yong Loo Lin School of Medicine, National University of Singapore (NUS), Singapore 119228.

## Abstract

Direct visualization and assessment of the iridocorneal angle (ICA) region with high resolution is important for the clinical evaluation of glaucoma. However, the current clinical imaging systems for ICA do not provide sufficient structural details due to their poor resolution. The key challenges in achieving high quality ICA imaging are its location in the anterior region of the eye and the occurrence of total internal reflection due to refractive index difference between cornea and air. Here, we report an indirect axicon assisted gonioscopy imaging probe with white light illumination. The illustrated results with this probe shows significantly improved visualization of structures in the ICA including TM region, compared to the current available tools. It could reveal critical details of ICA and expected to aid management by providing information that is complementary to angle photography and gonioscopy.

The trabecular meshwork (TM) in the iridocorneal angle (ICA) of the eye, plays a vital role in aqueous outflow. Aqueous humor flows continuously through the TM, at a rate of 2.75 μl/min approximately, to exit the anterior chamber of eye^1^. Abnormalities in TM can alter the aqueous humor flow rate, thereby increasing the intraocular pressure (IOP) which could subsequently lead to glaucoma. These abnormalities include marked loss of TM cells, fusion and thickening of the trabecular lamellae and deposition of sheath-derived plaques in comparison to age-matched normas. For this reason, direct high quality visualization of the TM is of potential diagnostic value.

There has been a lot of rapid advancement in the last decade in ophthalmology in the area of ICA imaging^2^,^3^,^4^,^5^. However, gonioscopic angle examination and current clinical imaging modalities such as EyeCam^6^,^7^, optical coherence tomography (OCT)^8^,^9^,^10^, Scheimpflug imaging (Pentacam-Scheimpflug)^11^, ultrasound biomicroscopy (UBM)^12^,^13^,^14^ and Orbscan scanning slit topography^15^ do not have the spatial resolution sufficient enough to resolve TM structures^16^. OCT is an effective tool to analyze quantitative parameters of the ICA. However, the OCT is not extensively used by clinicians as it is expensive tool, difficult to interpret and due to the fact that it only provides a single cross-sectional view of the eye. Pentacam using Scheimpflug principle allows noncontact quantification of the anterior chamber parameters^11^. The anterior curvature and posterior surface of the cornea can be analyzed by Orbscan scanning-slit topography^15^. However, direct visualization of ICA is not possible using either the Pentacam or Orbscan scanning slit topography^17^. UBM can be used for direct angle visualization, however, it is a contact procedure, expensive and has not been established as a common clinical procedure^2^.

The EyeCam^TM^ is a portable hand held photographic device used to image retina and the ICA. EyeCam has shown results comparable to gonioscopy^6^. EyeCam being a contact procedure and requiring supine positioning for imaging, could not find its use in routine clinical practice. Till date, gonioscopy remain the gold standard for assessing ICA in a clinical setting. It is popular among clinicians due to its low cost and the ability to indent the eye to visualize dynamic changes. However, neither gonioscopy nor EyeCam has sufficient imaging resolution to visualize TM region with high clarity.

There are also different indirect imaging systems developed with integration of gonioscopy with other techniques. In a notable study by Masihzadeh *et al.*, multiphoton gonioscopy was utilized to image TM structures^16^,^18^. The advantage of multiphoton gonioscopic imaging system lies in its ability to perform two photon autofluorescence and second harmonic generation to image TM region through gonioscope with better resolution and without using any fluorescence stains. However, multiphoton sources (lasers) are very expensive and are not used in routine clinical procedures. In 2015, McNabb *et al.* demonstrated complete 360° circumferential gonioscopic optical coherence tomography imaging of the iridocorneal angle^8^. However, high resolution structural information of the TM region was not possible by this method.

In this article, we report the first demonstration of an integrated gonioscopy concept named as axicon assisted gonioscopy. The proposed concept and method uses gonioscopy imaging approach by integrating Bessel Beam Microscopy (BBM) concept^19^,^20^. The proposed concept is explained through optical simulation which is followed by experimental demonstration using the developed system. The imaged ICA region of porcine eyes indicates the efficiency of the system and its clinical significance. A Comparison of ICA images taken by the newly developed system with gonioscopy and EyeCam, demonstrate improved resolution in documenting the TM, pigmentation and ciliary body base.

## Results

### Optical design and simulation of gonioscopic system

**Figure 1:**
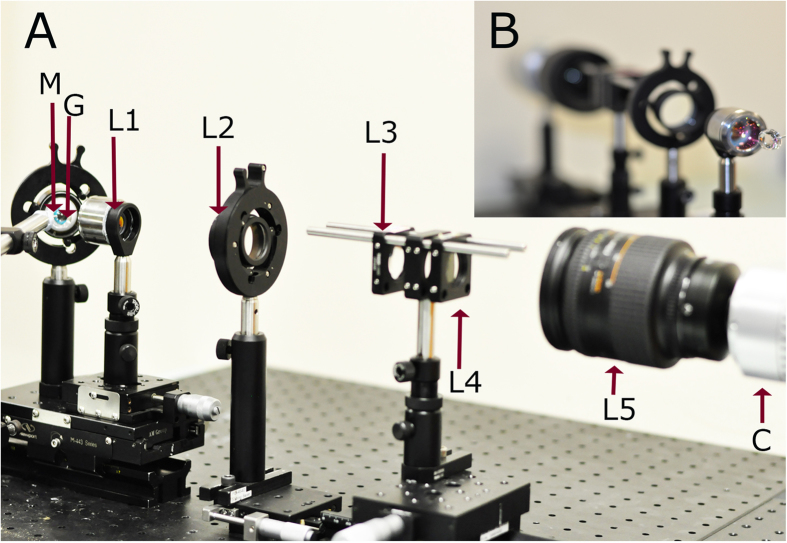
Optical setup of developed indirect gonioscopic imaging system. (**a**) Optical setup of the developed imaging system. (**b**) Inset, illustrating the front view of our system. [M: Eye model; G: Hoskins-Barkan Goniotomy lens; L1: objective lens; L2: tube lens; L3: convex lens; L4: axicon lens; L5: zoom lens; C: CCD].

**Figure 2:**
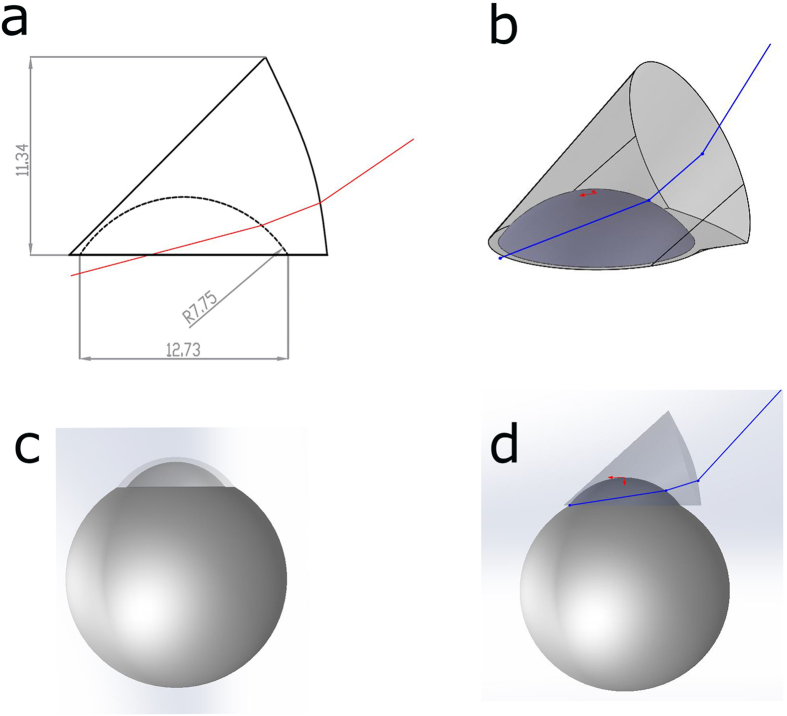
3D model of simulated gonio lens and eye model. (**a**) Cross section of simulated gonio lens. (**b**) 3D model of simulated gonio lens. (**c**) 3D model of simulated eye. (**d**) Illustration of placement of gonio lens on eye.

Figure 1 shows the optical setup of the proposed simple indirect white light gonioscopic imaging system (the schematic diagram is provided in the Supplementary Fig. S4). Here, the indirect gonioscopy imaging system is designed using a conventional gonioscopic contact lens (Hoskins-Barkan Goniotomy lens, adult 11 mm lens, Ocular instruments, WA, United States of America), as the imaging head. For optical simulation, a 3D model of gonioscopic lens is made using Zemax software by using the design specifications of Hoskins-Barkan Goniotomy lens. Figure 2 shows the 3D model of gonioscopic lens and eye. In Fig. 2, only the chief ray is illustrated. The parameters for the eye model and ray tracing of the imaging system are provided in the Supplementary material.

**Figure 3:**
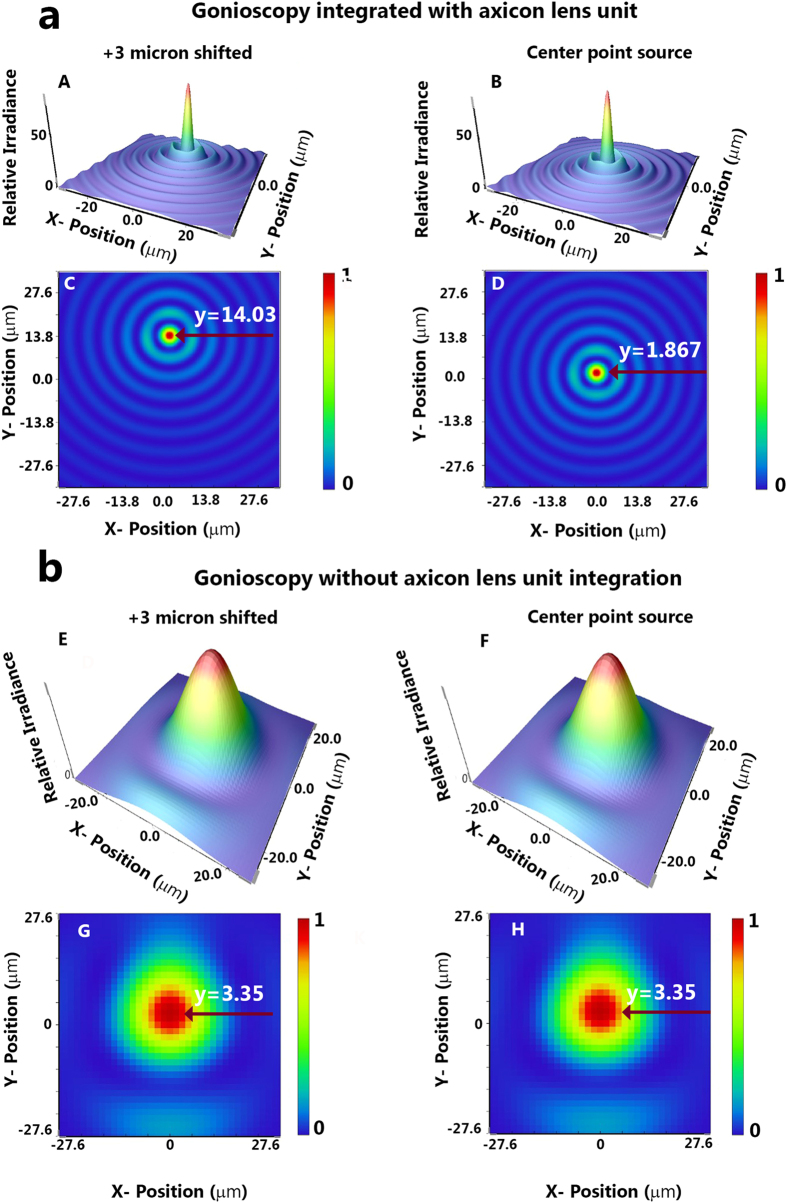
Resolution comparison for gonioscopy integrated with and without axicon lens unit. (**a**) Huygens PSF simulation by Zemax of our gonioscopy system integrated with axicon lens unit. (**b**) Huygens PSF simulation by Zemax of the gonioscopy system without axicon lens unit. (Here we have performed separate PSF simulation each for point sources at origin and after 3 μm shift in Y axis).

**Figure 4:**
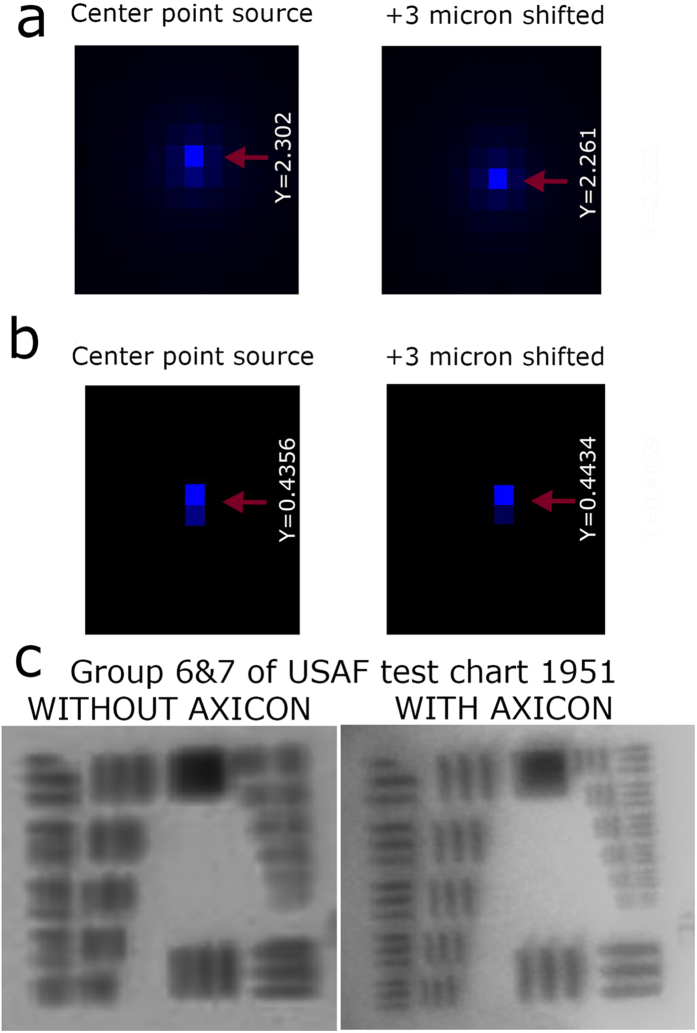
Illustration of PSF grid and USF chart imaging. (**a**) PSF grid simulation by Zemax of our gonioscopy system integrated with axicon lens unit. (**b**) PSF grid simulation by Zemax of the gonioscopy system without axicon lens unit. (**c**) USAF test chart 1951, imaged by our gonioscopy system without and with axicon lens unit.

The results of 3D Huygen point spread function (PSF) and the PSF grid for gonioscopy imaging system integrated with and without axicon lens unit are shown in Figs 3 and 4. We performed the simulations with two point sources separated by 3 μm. For the gonioscopy imaging system integrated with axicon lens unit, improvement in resolution is evident from Huygen PSF, which showed a distinct separation of the 3 μm point sources. However, it is also evident from Fig. 3 that when axicon is not used there is clear overlap of the two point sources. The difference between the gonioscopy imaging system with and without the axicon lens unit was further compared with result of PSF grid simulation, as shown in Fig. 4.

For theoretical modelling, we used a plano convex lens (F = 25 mm) in combination with a gonio lens as the imaging head. However, for performing the experiments, we utilized LSM03 (4.6X, VIS OCT scan lens, Thorlabs, USA) instead of plano convex lens. Therefore, we further performed an experimental comparison of both systems by imaging a USAF chart through gonio lens. The result is shown in Fig. 4c. The USAF resolution chart 1951, group 7 element 4 (2.76 μm) was resolved for gonioscopy system integrated with the axicon lens unit. On the other hand, the gonioscopic system without the axicon lens unit only resolved group 6 element 2 (16 μm) (Fig. 4c). Thus, it is clearly evident from these results that the performance of gonioscopy system in terms of spatial resolution is improved with the integration of axicon lens unit.

### ICA image of demo eye

**Figure 5:**
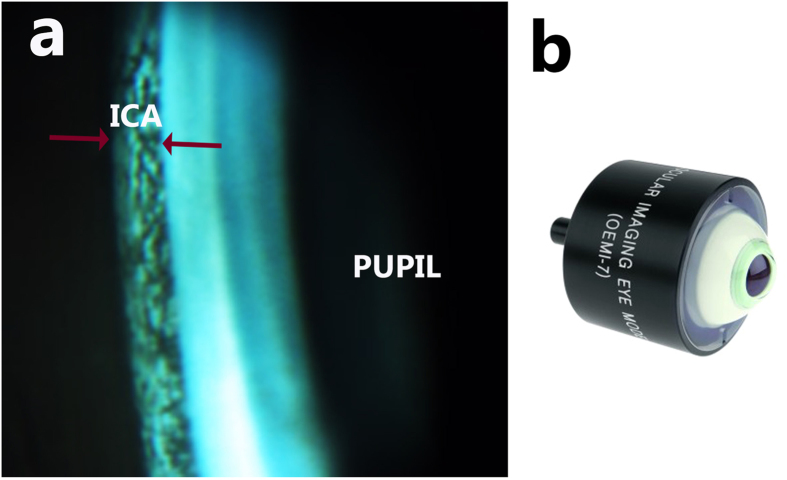
Illustration of iridocorneal angle of eye model imaged by the developed system. (**a**) ICA region of model eye imaged by the imaging system. (**b**) Photograph of demo eye (OEM1-7, Ocular Instruments Inc., Bellevue, WA, USA). The red arrow highlight the ICA region.

Functionality of the imaging system was first tested on an ocular imaging eye model (OEM1-7, Ocular Instruments Inc., Bellevue, WA, USA). Gonioscopic lens was placed onto the eye model interfacing with coupling gel (Vidisic gel). The result is shown in Fig. 5a. Figure 5b shows the image of the demo eye model.

### TM region of porcine eye visualized by the developed probe

**Figure 6:**
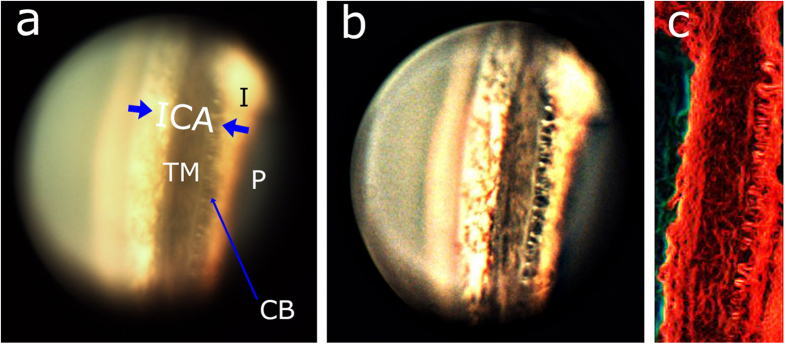
Acquired image of iridocorneal angle of porcine eye imaged by the developed system. (**a**) Raw TM region image of porcine eye. (**b**) Processed TM region image of porcine eye. (**c**) Edge detected image allowing structures inside TM region to be visualized. [ICA: iridocorneal angle; P: Pupil; I: Iris; CB: Ciliary body, TM: Trabecular meshwork.]

The ICA of the porcine eyes imaged with our system is shown in Fig. 6. Figure 6a is the acquired raw image. Raw image has structures slightly false colored since the light source used to illuminate has yellow tint and not perfectly white. The processed image is shown in Fig. 6b. It is evident that the different regions in the ICA of the porcine eye are distinctly visible in the image. Close to the iris, there is a simple organization of ciliary body base circumferentially distinct in a discontinuous bundle like pattern. In this zone, fibers are longer and reach the sclera and the scleral spur. It is also evident that the ciliary body is highly pigmented. This observation is corroborated by the earlier studies which indicated higher pigmentations in porcine ciliary body as compared to ciliary body of human eyes^21^.

The TM region is very distinct in the image (Fig. 6b). However, TM meshwork structures are further highlighted by performing edge detection on the image. This result is shown in Fig. 6c. Pigmentation in the angular region is also clearly evident in Fig. 6b. It is important to note that the imaging was performed on enucleated porcine eyes, and we therefore assume the image quality will be even better for live animal due to less or no corneal haze.

### Comparison with other direct ICA imaging methods

**Figure 7:**
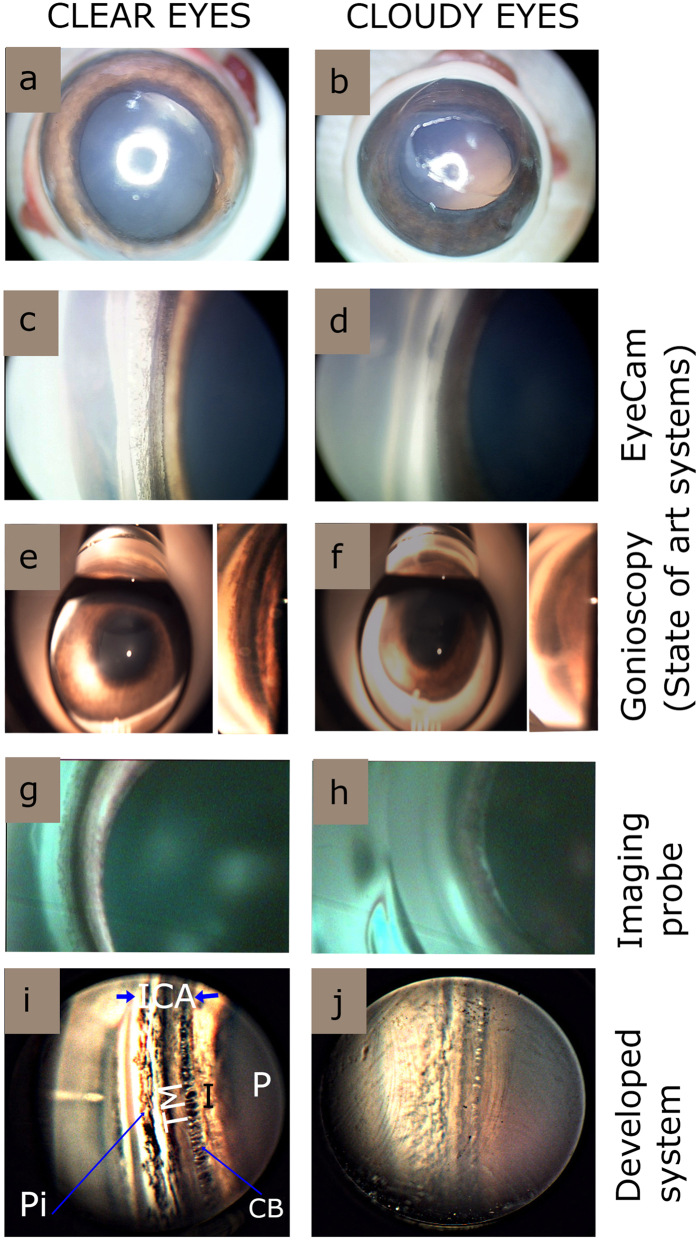
Comparison of various imaging system for imaging iridocorneal angle for clear and cloudy eyes. (**a**,**b**) Example photographs of clear and cloudy eyes respectively. (**c**,**d**) Still images by EyeCam (Clarity Medical systems, Pleasanton, CA, USA). (**e**,**f**) Still images through Gonioscope (Latina 5 Bar SLT, single mirror, Ocular instruments, WA, USA). (**g**,**h**) Still images aptured using dual modality hand held probe. (**i**,**j**) Still images taken by our imaging system. Note for (**e**–**h**) the angle regions of gonioscopy images are digitally zoomed and highlighted as separate insets. [ICA: iridocorneal angle; P: Pupil; I: Iris; Pi: Pigmentation CB: Ciliary body, TM: Trabecular meshwork.]

Figure 7 shows the comparison of ICA of Porcine eyes imaged using the newly developed system with the state of the art imaging systems such as EyeCam and Gonioscopy. The amount of clouding (haze) of the cornea is known to affect the image quality drastically. Due to this reason, imaging was performed on clear and mildly cloudy porcine eyes with all the imaging systems, for comparative purposes. It is evident from the Fig. 7 that, irrespective of the different imaging systems used, the structures of the ICA imaged for clear eyes were more distinct than cloudy ones. The details of the EyeCam images were better than gonioscopic images. Wide field camera of the EyeCam also allows to get a larger view of iridocorneal angle region as compared to gonioscopy images (Fig. 7). However, it is clear ICA imaged with our system is much better than EyeCam and gonioscopy. Notably, even for cloudy eyes our system showed better image clarity (Fig. 7j). Moreover, fine structural details of pigmentation, TM, ciliary body can be visualized by our system.

Further, we also compared the results with an integrated flexible handheld wide angle probe which was recently developed in our research centre for imaging and documenting ICA^17^ (Fig. 7g,h). Though the probe is flexible and can image large area using an endoscopic CCD camera as compared to other imaging systems, the resolution is poor compared to the proposed new axicon assisted gonioscopy. All the above mentioned results confirms the improved quality of ICA imaging which can be achieved by our system as compared to the state of the art systems as well as those reported in literatures.

### Illustration of pigmentation in the ICA with clarity

Assessment of pigmentation in the TM is of major significance to glaucoma diagnosis^22^,^23^,^24^. (Porcine eyes are generally known to have higher pigmentation than human eyes^25^.) The pigmentation pattern of an individual angle is dynamic over time. Heavy pigmentation of the TM should indicate pigment dispersion or exfoliation syndrome. In addition, a line of pigment deposition anterior to the Schwalbe line is often present in exfoliation syndrome (Sampaolesi line). Moreover, studies have also revealed that pigmentation can affect or introduce challenges for glaucoma treatment such as laser trabeculoplasty^26^. Other conditions that cause increased anterior chamber angle pigmentation include malignant melanoma, trauma, surgery, inflammation, angle closure, and hyphema.

**Figure 8:**
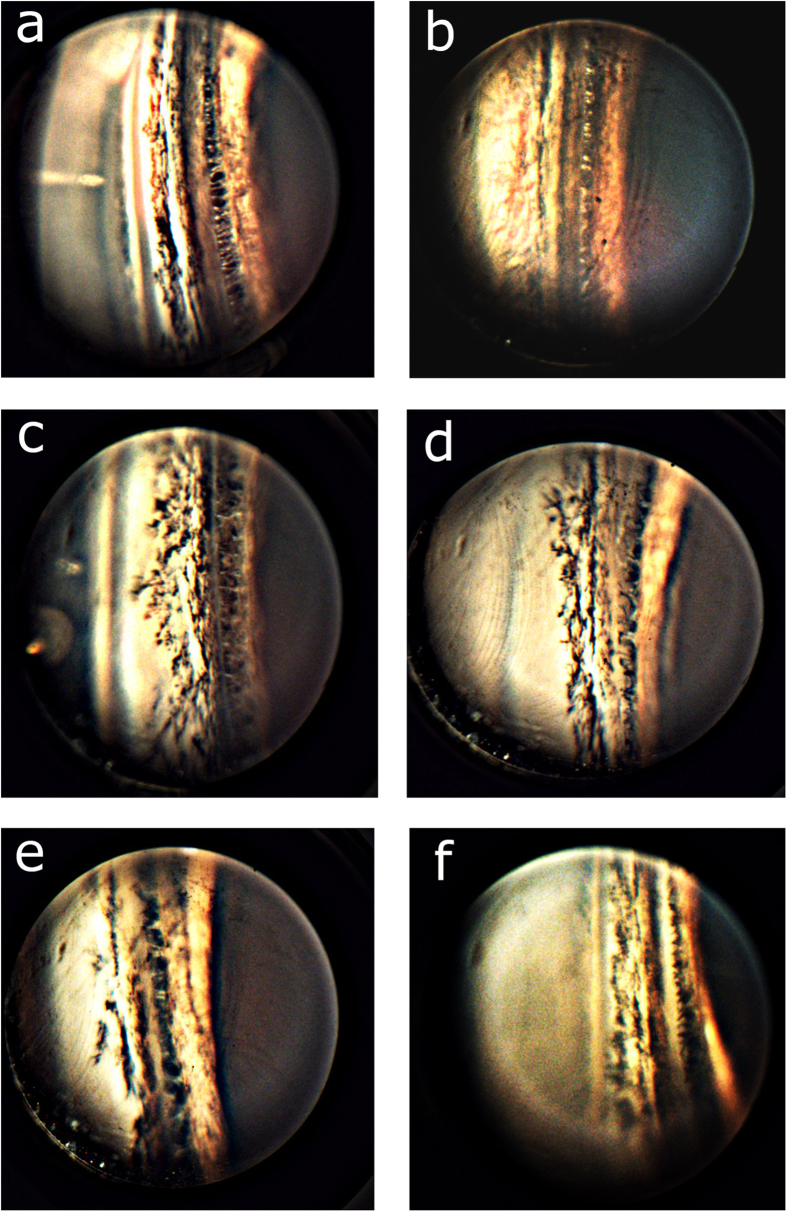
Illustration of iridocorneal angle images for different porcine eyes with varying trabecular meshwork pigmentation.

However, there are only few relevant studies which statistically analyzed the amount of pigmentation in the TM region^27^,^28^,^29^. Currently available imaging systems can neither provide new insight nor quantify the pigment level of TM^27^. For this reason, we have demonstrated the potential of our system by illustrating better quality porcine eye images with different pattern of pigmentation. The result is shown in Fig. 8. It is evident from Fig. 8, that the pigmentation have better contrast. We believe the high contrast imaging of ICA region would be useful to perform further statistical studies to get quantitative information regarding pigmentation.

## Discussion

In vision sciences research, porcine eyes were often used as an *ex vivo* animal model because of similarity in its morphology to the human eye^30^,^31^. In addition, porcine eyes are also generally used as animal model eyes for validation in a variety of studies such as R & D on glaucoma^32^, in neuroretinal studies^33^, cataract surgery^34^, corneal transplant studies^35^ and in aberrometry studies^36^. In our study, we have used the porcine eye as the test eye samples because of its resemblance to the human eye and due to easy availability.

ICA imaging systems must have resolution in the range of 1 to 5 μm^16^ to resolve TM region. However, the existing clinical white light gonioscopic systems do not have this above mentioned spatial resolution to resolve the structural details of TM. This key drawback limits their efficiency to visualize critical details of ICA.

In summary, we have demonstrated for the first time to the best of our knowledge, an axicon assisted white light indirect gonioscopic system which can image the ICA with spatial resolution down to 3 μm. This imaging system is thus capable of direct imaging of ICA with better structural clarity as compared to current imaging system such as gonioscopy, OCT and EyeCam. Our gonioscopic system may pave the way for future ICA imaging.

## Methods

### Preparation of Sample

Enucleated porcine eyes were obtained from abattoir. To avoid corneal haze which would reduce image quality, porcine eyes were imaged within 5 to 6 hours of death. The *ex vivo* samples were transported on ice (approximately −4 °C) to the laboratory to maintain their freshness. Before imaging, the extraocular tissues such as conjunctiva and lacrimal gland were removed from the samples. Each sample was fixed on to a custom eye holder, which was mounted on a translational stage with micrometer accuracy. A sample size of 50 porcine eyes were used for the study. The experiment was conducted in accordance to the regulations of Agri-Food & Veterinary Authority of Singapore and Nanyang Technological University’s regulations on biosafety.

### Gonioscopy

Hoskins-Barkan Goniotomy lens (adult 11 mm lens, Ocular instruments, WA, USA) was used in this study. The thickness of the gonioscopic lens is 1 cm. The gonioscopic lens was fixed to an optical post and secured to optical table. A thin layer of ophthalmic gel (Vidisic Gel, Bausch & Lomb, NY, USA) was applied to the gonioscopic lens before placing it on the eye, to provide lubrication and to reduce the refractive index mismatch. The porcine eye (with cornea in centre) was then placed gently on to the developed gonioscopic lens based imaging system to image the TM region in the ICA.

Additionally using Latina 5 Bar SLT, (single mirror, Ocular instruments, WA, USA) static gonioscopy was also performed on porcine eyes under white light. Vidisic gel was used as the lubricant. Gonioscopy was performed on clear as well as mildly cloudy porcine eyes to compare the image quality. The images were recorded using a colour CCD camera.

### EyeCam

EyeCam (Clarity Medical systems, Pleasanton, CA) imaging was also performed on porcine samples (n = 4). The images were taken by a trained technician at Singapore Eye Research Institute, who is specialized on EyeCam imaging and had basic knowledge of angle anatomy and structures. Using foot pedal, illumination was adjusted to avoid overexposure. Clear still images of the ICA were then recorded with the probe being placed at the opposite limbal region.

### In-house developed handheld imaging probe for iridocorneal angle

The flexible handheld probe has an imaging sensor located at the central axis of the probe and has a variable resolution at different depths which is optimized for recording the ICA of the eye^17^. The imaging probe was placed near the limbal region of the porcine cornea to image the opposite ICA through vidisic gel. The imaging probe has LEDs integrated to provide illumination.

### Optical Setup

In the gonioscopic setup shown in Fig. 1, a white light source (Correct Shimadzu FA-150EN Fiber Illuminator, Japan) was used for illumination. (3D model and the schematic diagram is also provided in the Supplementary Fig. S4.) The imaging head is a gonioscopic lens (Hoskins-Barkan Goniotomy adult 11 mm lens; Ocular Instruments, WA, USA). Through the gonioscopic lens, ICA region of the eye was imaged by a microscopic objective lens (LSM03 - 4.6X, VIS OCT scan lens, Thorlabs, USA). The long working distance of the objective lens (25 mm) and it’s large field of view enabled the integration of the microscopic lens with a gonioscopic lens. The light from the microscopic objective was then collected via a tube lens (ITL 200, Thorlabs). A convex lens (LB-1844-A, Thorlabs) was placed at a focal length distance from the focal plane of the tube lens. An axicon lens was placed in close proximity of convex lens. The combination of convex lens and axicon lens was known to transform the wavefront of a point source into a Bessel beam, which has the special property to propagate without diffracting. A beam passing through axicon lens was captured by the CCD camera (EXi Aqua Bio-Imaging Microscopy Camera, QImaging, Canada). Lateral resolution of the imaging system is around 3 μm. Further, optical ray tracing of the imaging system is detailed in the Supplementary Fig. 3. Additionally information about basic BBM configuration is further detailed in the Supplementary material.

Acquired images were post processed using ImageJ software. In order to enhance the image quality, noise reduction filtering and thresholding was applied to the captured images. Additionally, edge detection was also performed to highlight the structures in the images.

## Additional Information

**How to cite this article**: Perinchery, S. M. *et al.* High resolution iridocorneal angle imaging system by axicon lens assisted gonioscopy. *Sci. Rep.* **6**, 30844; doi: 10.1038/srep30844 (2016).

## Supplementary Material

Supplementary Information
