# Supplementary material for: High resolution iridocorneal angle imaging system by axicon lens assisted gonioscopy
Source: Sci Rep. 2016 Jul 29;6:30844. doi: 10.1038/srep30844 (PMC4965779; doi:10.1038/srep30844)
Supplement: Supplementary Information [file srep30844-s1.pdf]

Supplementary Material

High resolution iridocorneal angle imaging system by axicon lens assisted gonioscopy

Sandeep Menon Perinchery[1,2] , Anant Shinde[1,2] , Chan Yiu Fu[1,2], Hong Xun Jie Jeesmond[1,2], Mani Baskaran[3], Tin Aung[3,4] and Vadakke Matham Murukeshan[1,2*]

[1]School of Mechanical and Aerospace Engineering, Nanyang Technological University, Singapore 639798

[2]Centre for Optical and Laser Engineering, 50 Nanyang Avenue, Singapore 639798

[3]Singapore Eye Research Institute (SERI) and Singapore National Eye Centre (SNEC), 11 Third Hospital Avenue, Singapore 168751, Singapore

[4]Yong Loo Lin School of Medicine, National University of Singapore (NUS), Singapore 119228

*Address for Correspondence: mmurukeshan@ntu.edu.sg

1. BBM configuration

Supplementary Fig. S1A shows 1951 USAF test chart imaged using classical system consisting of an objective lens (LSM03, N.A: 0.055) and a tube lens. It is evident that group 6 element 4 of USAF test chart is the clearly resolvable, which has a line thickness of 5.52 µm.

In **Supplementary Fig. S1B**, the optical configuration has a plano convex lens (f=50 mm) after the tube lens (no axicon lens). It is important to note that the plano convex lens focus is placed exactly at the focus position of tube lens. This configuration results in the increased magnification (2.66X) compared to classical system. However from **Supplementary Fig. S1B** it is evident that the resolution is not improved. Group 6 element 4 of USAF test chart is the clearly resolvable, which has a line thickness of 5.52 µm. This result is similar to the result shown in **Supplementary Fig. S1A**.

For the BBM system, we have introduced an axicon lens after the plano convex lens.

The minimum resolvable distance between two point sources for BBM is given by d_{min} (Snoeyink C *et al*[1])

$$d_{min} = f_{obj} \frac{2.405\, \lambda\, D}{2\pi\alpha(n-1)C}$$

Where,

f_{obj} = 2.5 cm (effective focal length of objective)

λ = 540 nm (wavelength)

D=1 (degree to which the PSF is focused or defocused)

α = 2° (surface angle of axicon)

n =1.46 (refractive index of axicon, UV fused silica)

C = 16 cm (effective distance from axicon to the camera)

From the above equation the value of d_{min} is calculated for our BBM configuration, which is 2.01 µm. This result is also corroborated by imaging 1951 USAF test chart through BBM base system. **Supplementary Fig. S1C** shows USAF chart group 7 element 6 resolved, which has line thickness of 2.19 µm. Improvement in resolution is evident theoretically and by experiment for BBM system.

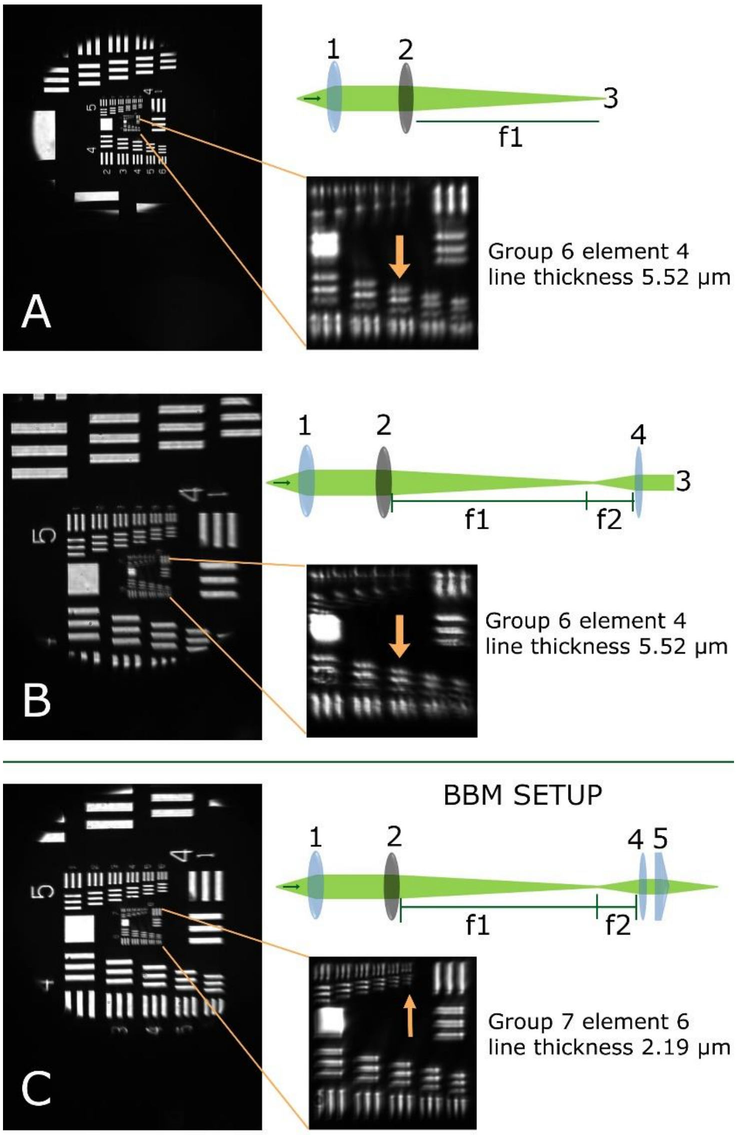

Supplementary Figure S1| Comparison of resolution. USAF 1951 test chart imaged through **a**, classical simple imaging system with objective and tube lens. **b**, classical imaging system with an additional lens between tube lens and camera. **c**, BBM system. [1: LSM03 Thorlabs; 2: tube lens with f2=148 mm; 3: camera, 4: plano convex lens with f1=50 mm; 5: axicon lens;]. Note: schematic diagram also shown for each optical configuration.

[The images are taken by **Sandeep Menon Perinchery**]

For the BBM system, since we have introduced an axicon lens after the plano convex lens. This results in demagnification in the size of image by 0.86X (**Supplementary Fig. S1C**) when compared to image captured with only plano convex lens of similar specifications (**Supplementary Fig. S1B**). Even though image is demagnified for BBM configuration, it is evident from the **Supplementary Fig. S1C** that resolution is improved. These results clearly show the role of axicon lens in the improving resolution of the BBM system.

For these reasons we have used gonio lens in combination with the BBM configuration. The gonio lens integrated with BBM system shows improvement in resolution as compared to conventional gonioscope. (refer manuscript, **Fig. 4C**).

However, the reduction in the resolution for the gonioscope integrated BBM system when compared to the BBM system is primarily contributed by the gonio lens. There can be multiple reasons for this. Refractive index of gonio lens can be one contributing factor. Further, it has been earlier reported that due to the steep angle of incidence on the surface of the gonioscopic lens, significant Fresnel reflection and scattering is possible[2].

2. Eye model parameters and simulation

For designing an eye model, accurate information about the geometric and optical properties such as radius of the curvature and refractive index of the cornea are needed. Further, scientific parameters for the eye model used for the design and simulation are to be well defined. Hence, in order to design the eye model, the parameters were chosen from the previous reported eye simulation reported by Herbert *et al.*[3]. Parameters used in eye model

are given in **Supplementary Table T1**. Although there would be slight differences between patients, the variations would be usually within the allowed tolerance zone.

Supplementary Table T1| Parameters for relaxed eye model by Navarro *et al*. [3,4]

NO	Type	Radius r [mm]	Thickness d [mm]	Index *n*	Aspherical *k*
1	Cornea	7.72	0.55	1.367	-0.26
2	Anterior chamber	6.50	3.05	1.3374	0
3	Crystalline lens	10.20	4.0	1.420	-3.1316
4	Vitreous humor	-6.00	16.4	1.336	-1.0
5	Image	-12.0			

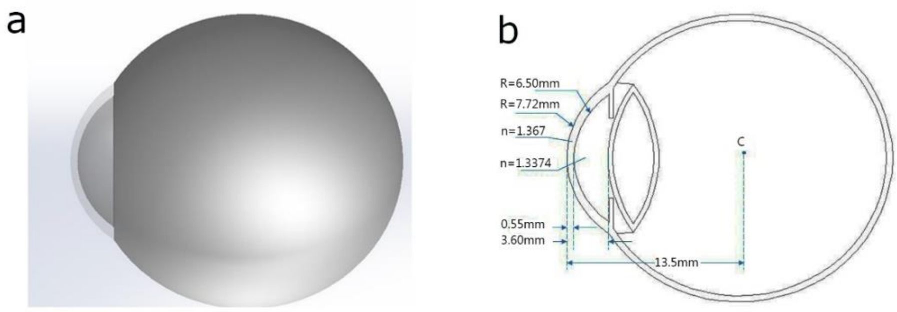

Supplementary Figure S2| Illustration of 3D shaded eye model and its cross sectional view.

[The images are done by **Chan Yiu Fu**]

Supplementary Fig. S2 shows the 3D shaded model and the cross sectional view of the simulated eye model.

3. Optical ray tracing

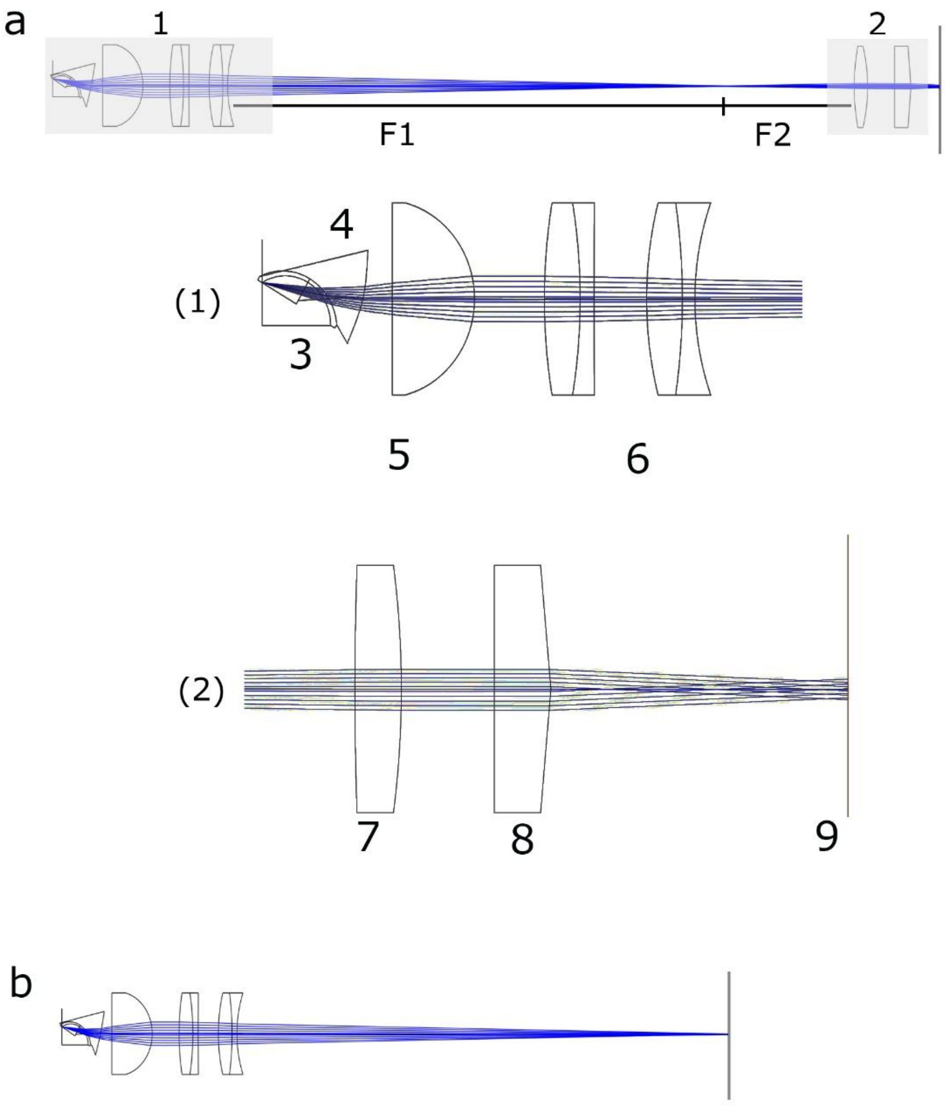

Supplementary Figure S3| Zemax optical ray tracing for the imaging system. a, Ray tracing for the developed indirect gonioscopic system with axicon lens unit. **b**, Ray tracing for indirect gonioscopic system without axicon lens unit. [3: Eye model; 4: Hoskins-Barkan Goniotomy lens; L2: objective lens; 6: tube lens; 7: convex lens; 8: axicon lens; 9: detector].

[The simulations are performed by **Chan Yiu Fu and Sandeep Menon Perinchery**]

In order to design the proposed indirect gonioscopy system with an axicon lens, we performed the optical ray tracing approach, using Zemax software. **Supplementary Fig. S3** shows the result obtained after implementing the ray tracing. For the optical simulation, we have used the gonioscopic lens model, which was earlier described in the manuscript (**Fig. 2**).

Though we have used a microscopic objective lens LSM03 (4.6X, VIS OCT scan lens, Thorlabs, USA) in the optical system, a Thorlabs 25mm convex lens was chosen for the simulation purpose. Additionally, a convex lens of 50mm focal length was used in combination with the axicon lens (**Supplementary Fig. S3)**. The convex lens focal point was placed exactly at the tube lens focal plane (148 mm). This gives a total distance of 198 mm from the tube lens to the convex lens. The rays that are collected exactly from the focal point of convex lens will propagate parallel to the optical axis. Therefore, ideally the placement (distance) of axicon from the convex lens is not critical. However, the axicon lens was placed close to the convex lens to subdue loss of light. Since the axicon has a large focal plane, position of detector is easy. Ray tracing was then executed by placing the source point at the anterior angle of the eye.

Further, we have also performed the optical ray tracing for gonioscopy system without the axicon lens unit. The result is shown in **Supplementary Fig. S3b**. In this configuration, the detector is placed exactly at the focus position of the tube lens (148mm). This was done to study the difference between the conventional gonioscopy systems with the proposed axicon lens unit integrated goinioscope. For both systems, Huygen PSF and PSF grid were analyzed (refer manuscript).

4. Schematic of optical setup

Supplementary Fig. S4 shows the 3D model and schematic diagram of the designed proposed axicon assisted gonioscopic system. This novel configuration with the simplicity of implementing axicon integration, can be easily operated by any clinicians. The unique combination of the gonioscopic lens (L3) and long working distance objective lens (L2) together with the Bessel Beam Microscopy concept (axicon unit L1 and A1) allow this

system to visualize the ICA with greater details and highly improved contrast and spatial resolution.

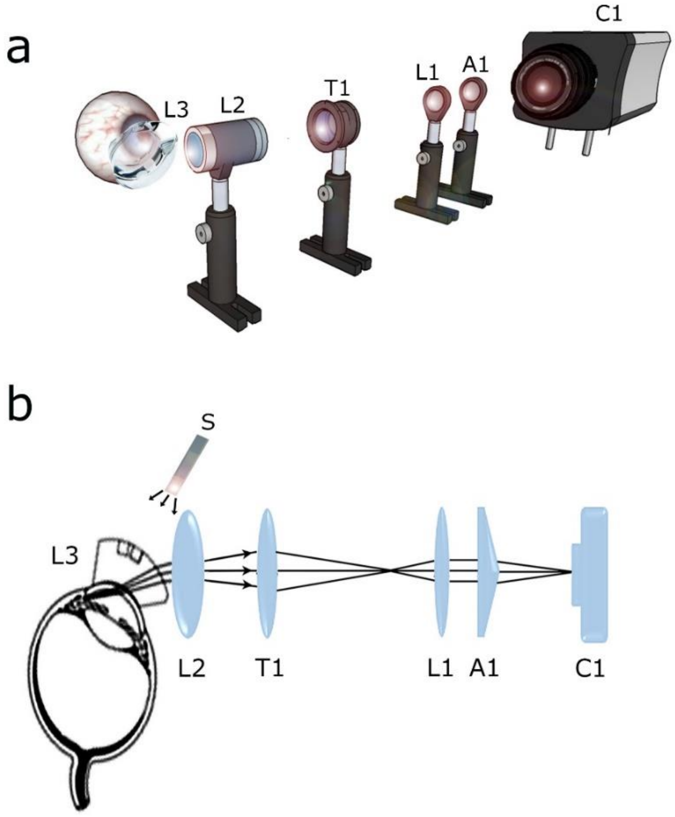

Supplementary Figure S4| Illustration of 3D model and Schematic diagram of developed Gonioscopy imaging. a, 3D model. **b**, Schematic diagram. L3: Hoskins-Barkan Goniotomy lens; L2: objective lens; T1: tube lens; L1: convex lens; A1: axicon lens; C1: color camera with zoom lens; S: white light source.

[The images are done by **Sandeep Menon Perinchery**]

While imaging the entire eye, the system has to be rotated, which is a limitation of our current system. However, this limitation can be overcome by upgrading the same system to a hand held portable imaging probe.

5. Placement of eye

The positioning of the eye is important for a good image. The eye with cornea at the center has to be gently placed onto the Hoskins-Barkan Goniotomy lens, as shown in **Supplementary Fig. S5**. The gonioscopic lens was fixed to an optical post and further secured to an optical table. Before the imaging begins, a thin layer of ophthalmic gel (Vidisic Gel, Bausch & Lomb, NY, USA) was applied to provide lubrication and to reduce refractive index mismatch.

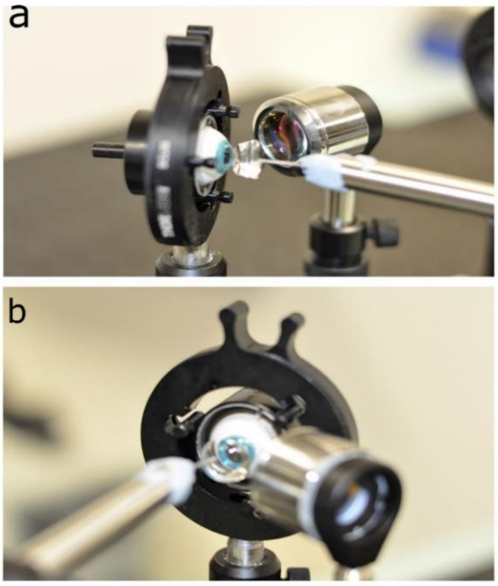

Supplementary Figure S5| Illustration of placement of eye onto gonioscopic lens. a, back view. **b**, front view.

[The images are taken by **Sandeep Menon Perinchery**]

6. Equipment safety and maintenance

If the luminous intensity of visible light is less than 10,000 Candela/m^2, no evaluation of retinal hazard is required according to the International Commission on Nonionizing

Radiation Protection act[5]. The white light source used in our study had maximum luminous intensity of 6000 mcd. This is well within the maximum permissible exposure (MPE) limit. The maintenance of the gonioscope imaging system include cleaning the gonioscopic lens using disinfectant solutions (e.g., Approved by FDA, DGHM), which can be used in accordance with label instructions given by the disinfectant manufacturer to clean the gonioscopic lens. Few drops of low foaming mild soap with neutral pH (7.0) detergent formulated for medical instruments on a moistened cotton ball can also be used to gently clean the gonioscopic lens. Thus, it does not require specialized cleaning solutions or protocol. Notably, the gonioscopic lens attached to the imaging arm is easily removable which reduces the possibility for infection.

References

1 Snoeyink, C. Imaging performance of Bessel beam microscopy. Opt Lett. **38**, 2550-2553 (2013).

2 Masihzadeh, O., Ammar, D. A., Kahook, M. Y., Gibson, E. A. & Lei, T. C. Direct trabecular meshwork imaging in porcine eyes through multiphoton gonioscopy. J Biomed Opt. **18**, 036009-036009-5 (2013).

3 Gross, H., Blechinger, F. & Achtner, B. *Handbook of Optical Systems*. Vol. 4 28-30 (Wiley, 2008).

4 Navarro, R., Santamaría, J. & Bescós, J. Accommodation-dependent model of the human eye with aspherics. *J. Opt. Soc. Am. A*. **2,** 1273-1280 (1985).

5 ICNIRP. Guidelines on limits of exposure to broad-band incoherent optical radiation (0.38 to 3 microM). International Commission on Non-Ionizing Radiation Protection. *Health physics*. **73,** 539-554 (1997).
